# Structural Analyses of *Avocado sunblotch viroid* Reveal Differences in the Folding of Plus and Minus RNA Strands

**DOI:** 10.3390/v6020489

**Published:** 2014-01-29

**Authors:** Clémentine Delan-Forino, Jules Deforges, Lionel Benard, Bruno Sargueil, Marie-Christine Maurel, Claire Torchet

**Affiliations:** 1 Sorbonne Universités, Université Pierre et Marie Curie UPMC Univ Paris 06, Centre National de la Recherche Scientifique, FRE3207, Laboratoire ANBIOPHY, Fonctions et Interactions des Acides Nucléiques, 75005 Paris, France; E-Mails: clementine.delanforino@gmail.com (C.D.-F.); marie-christine.maurel@upmc.fr (M.-C.M.); 2 Centre National de la Recherche Scientifique, Unité Mixte de Recherche UMR8015, Laboratoire de Cristallographie et RMN Biologiques, Université Paris Descartes, 4 avenue de l’Observatoire, 75270 Paris Cedex 06, France; E-Mails: jules.deforges@gmail.com (J.D.); bruno.sargueil@parisdescartes.fr (B.S.); 3 Centre National de la Recherche Scientifique, Unité Mixte de Recherche UMR8226, Laboratoire de Biologie Moléculaire et Cellulaire des Eucaryotes, Institut de Biologie Physico-Chimique, 75005 Paris, France; E-Mail: lionel.benard@ibpc.fr; 4 Sorbonne Universités, Université Pierre et Marie Curie UPMC Univ Paris 06, Unité Mixte de Recherche UMR8226, Laboratoire de Biologie Moléculaire et Cellulaire des Eucaryotes, Institut de Biologie Physico-Chimique, 75005 Paris, France

**Keywords:** *Avocado sunblotch viroid*, Ribozyme, RNA structure, SHAPE, *Avsunviroidae*

## Abstract

Viroids are small pathogenic circular single-stranded RNAs, present in two complementary sequences, named plus and minus, in infected plant cells. A high degree of complementarities between different regions of the RNAs allows them to adopt complex structures. Since viroids are naked non-coding RNAs, interactions with host factors appear to be closely related to their structural and catalytic characteristics. *Avocado sunblotch viroid* (ASBVd), a member of the family *Avsunviroidae*, replicates via a symmetric RNA-dependant rolling-circle process, involving self-cleavage via hammerhead ribozymes. Consequently, it is assumed that ASBVd plus and minus strands adopt similar structures. Moreover, by computer analyses, a quasi-rod-like secondary structure has been predicted. Nevertheless, secondary and tertiary structures of both polarities of ASBVd remain unsolved. In this study, we analyzed the characteristic of each strand of ASBVd through biophysical analyses. We report that ASBVd transcripts of plus and minus polarities exhibit differences in electrophoretic mobility under native conditions and in thermal denaturation profiles. Subsequently, the secondary structures of plus and minus polarities of ASBVd were probed using the RNA-selective 2'-hydroxyl acylation analyzed by primer extension (SHAPE) method. The models obtained show that both polarities fold into different structures. Moreover, our results suggest the existence of a kissing-loop interaction within the minus strand that may play a role in *in vivo* viroid life cycle.

## 1. Introduction

Viroids, a class of plant pathogens, are single-stranded, covalently closed circular RNA molecules with a chain length between 246 and 401 nt [1]. Since viroid RNAs do not code for any protein, the different phases of their life cycle, such as cellular transport, replication, and induction of pathogenicity, depend entirely on the capability of the RNA molecule to interact with cellular host factors. Thus, the determination of viroid secondary and tertiary structures of both strand polarities is crucial to better understanding how these small RNAs are able to use host machineries. On the basis of sequence homologies and self-cleavage ability, viroids have been classified into two families, *Pospiviroidae* and *Avsunviroidae* [2,3,4,5]. The secondary structure of *Potato spindle tuber viroid* (PSTVd), the type species of *Pospiviroidae*, which has been extensively studied by various biochemical methods, adopts a rod-like structure in solution [6,7,8,9]. In contrast to *Pospiviroidae*, the four members of *Avsunviroidae* including *Avocado sunblotch viroid* (ASBVd), *Peach latent mosaic viroid* (PLMVd), *Chrysanthemum chlorotic mottle viroid* (CChMVd) and *Eggplant latent viroid* (ELVd), are able to self-cleave, in both polarity strands, through a hammerhead ribozyme and replicate via a symmetric rolling circle pathway [10,11].

To date, the secondary structure of only one member of *Avsunviroidae*, PLMVd, has been determined in solution [12,13]. Using various approaches, it has been shown that (+) and (−) polarities of PLMVd adopt branched secondary structures [12,13,14]. Interestingly, structural differences between the two strands were identified, especially the formation of a kissing-loop in the (+) strand [12,14]. The possibility of a similar interaction in CChMVd, that also presents predicted branched secondary structures, has been suggested [12,15]. Regarding ASBVd, a quasi-rod-like structure has been predicted [16], but to date, neither the secondary structures of (+) nor (−) strands of ASBVd have been studied experimentally. Both polarities of ASBVd share many biological properties; therefore, one intriguing question is whether or not they adopt similar structures. Interestingly, purified (−) and (+) dimeric RNA transcripts are able to follow different folding pathways to reach their active self−cleaving structures. Indeed, the (−) strand of ASBVd undergoes self-cleavage by a double–hammerhead structure during *in vitro* transcription and by a single-hammerhead structure after purification of dimeric RNAs, whereas the (+) strand requires the more stable double-hammerhead structure to mediate self-cleavage during and after *in vitro* transcription [17]. Furthermore, (+) strands are present in a significantly higher amount in infected plant cells compared to (−) strands, suggesting a difference in replication efficiency and/or stability between the two polarities of ASBVd [18,19]. The aim of this study was to analyze the structure of both polarities of ASBVd to understand how it may influence viroid functions. Here, we report that *in vitro* time course experiments of self-cleavage show significant differences between ASBVd (−) and (+) monomeric RNAs. Subsequently, biophysical analyses of the characteristic of both strands demonstrate differences in electrophoretic mobility under native conditions and in thermal denaturation profiles. Finally, the secondary structures of (+) and (−) strands of ASBVd have been probed using the selective 2'-hydroxyl acylation analyzed by primer extension (SHAPE) method [20,21,22]. The models obtained suggest that the strands of both polarities fold into different structures, and that the (−) strand can adopt a tertiary structure, while we were unable to identify any tertiary interaction for the (+) strand.

## 2. Results and Discussion

### 2.1. Monomeric ASBVd (+) and (−) RNAs Containing the Full Hammerhead Ribozyme, Exhibit Different Self-Cleavage Time Course Experiments

**Figure 1:**
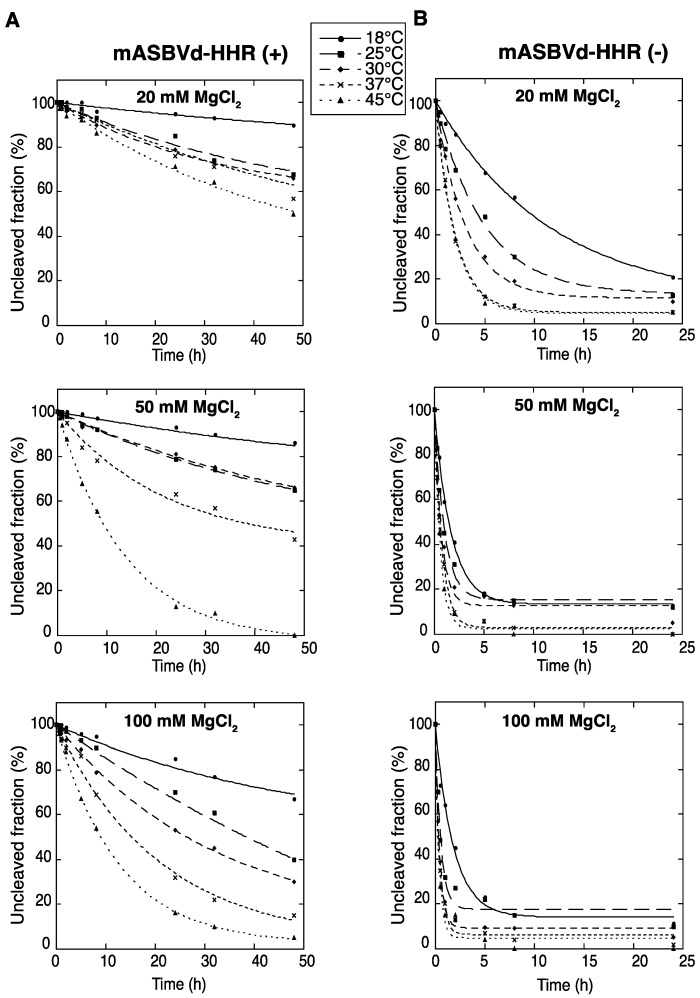
Self-cleavage kinetics of purified monomeric ASBVd-HHR (+) (**A**) and ASBVd-HHR (−) (**B**) transcripts. Self-cleavage reactions were carried out at 18 °C, 25 °C, 30 °C, 37 °C and 45 °C in buffers containing either 20 mM, 50 mM or 100 mM MgCl_2_. Aliquots were removed at different time up to 48 h for ASBVd (+) (panels **A**) and 24 h for ASBVd (−) (panels **B**), quenched with an excess of stop solution and separated on 10% polyacrylamide gels. After ethidium bromide staining, the percentage of uncleaved molecules was quantified using the ImageJ Software [25]. Quantitative data were fitted to multiexponential curves with the KaleidaGraph program (Synergy Software, Reading, PA, USA).

To evaluate the catalytic activity of monomeric ASBVd (+) and (−) encompassing the full hammerhead ribozyme (HHR), self-cleavage time course experiments of mASBVd-HHR (+) and mASBVd-HHR (−) RNAs were performed as indicated in the Experimental Section. Similar profiles of self-cleavage were observed whether 10 mM sodium cacodylate (pH 7.2) or 45 mM Tris-HCl (pH 7.5) was used as a buffer. With 20 mM MgCl_2_, mASBVd-HHR (+) remained essentially intact (95%) after 5 hours of incubation at 37 °C, whereas 88% of the mASBVd-HHR (−) was cleaved under the same conditions. The fraction of uncleaved mASBVd-HHR (+) molecules reached 20% at 50 mM MgCl_2_, only at 45 °C and after 20 hours of incubation (Figure 1A, note that some RNA degradation was observed after 24 hours in 100 mM MgCl_2 _at 45 °C). In contrast, similar self-cleavage time course experiments were observed for the mASBVd-HHR (−) at 50 mM and 100 mM MgCl_2 _at each temperature, with the fraction of uncleaved molecules at the end-point of the reaction being less than 20% in all cases (Figure 1B). Altogether, these results indicate that the majority of monomeric ASBVd-HHR (+) molecules are not able to adopt a catalytically active conformation compared to mASBVd-HHR (−) RNAs. This difference in the self-cleavage characteristic of (+) and (−) monomeric RNA transcripts is consistent with the difference previously observed with the dimeric transcripts [17]. It is worth noting that it has been shown that self-cleavage of the hammerheads of CChMVd and PLMVd as well as of satellite viruses of *Tobacco ringspot virus* (sTRSV) and *Chicory yellow mottle virus* (sCYMV) were greatly favored by tertiary interactions between the peripheral regions that stabilize the active conformation [23,24]. Similarly, the significant differences observed in catalytic activities for the (+) and (−) polarities of ASBVd could at least partially originate from interactions with sequence elements outside of the conserved catalytic core.

### 2.2. TGGE and UV Melting Analyses of the (+) and (−) Polarities of ASBVd RNAs

**Figure 2:**
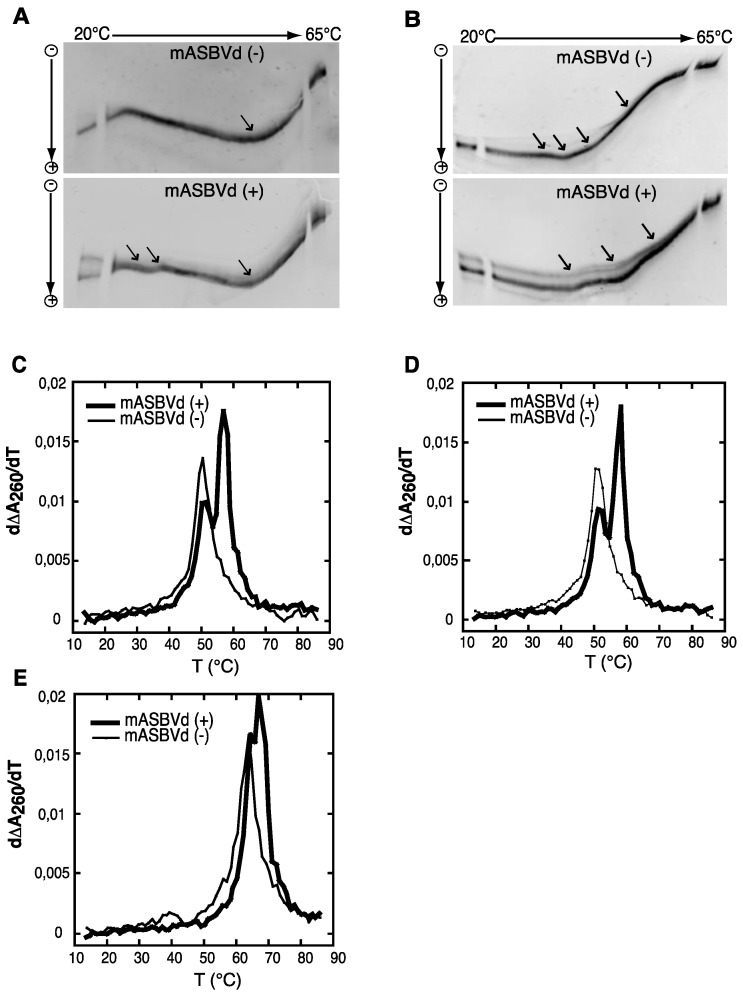
Temperature gradient gel electrophoresis (TGGE) and melting curves of mASBVd (+) and mASBVd (−) transcripts. (**A**) TGGE analysis performed by native 8% PAGE in 0.2× TBE buffer. (**B**) TGGE analysis performed by native 8% PAGE in 0.2× TB buffer containing 20 μM magnesium acetate. Migration was monitored between 20 °C and 65 °C. The arrows denote the transition temperatures. Derivative absorbance melting profiles were determined either in 150 mM KCl and 10 mM sodium cacodylate (pH 7.2) (**C**) or with, in addition, 100 μM MgCl_2_ (**D**) or 1 M NaCl (**E**). The first derivative profiles (d∆A_260_/dT) are shown with a thick curve for mASBVd (+) and a thin curve for mASBVd (−).

Both polarities of monomeric ASBVd transcripts were analyzed by temperature gradient gel electrophoresis analysis (TGGE), which allows the detection of co-existing alternative structures for a single type of RNA, over a range of temperatures (for review see [26]). It is important to note that for these analyses, purified unit-length transcripts of both polarities of ASBVd were used, *i.e.*, similar to monomeric species obtained after self-cleavage of multimeric forms and consequently different from the one used previously for the self-cleavage time course experiments (see Experimental Section). The TGGE denaturation profiles obtained for mASBVd (+) and mASBVd (−) transcripts, with a linear temperature gradient between 20 °C and 65 °C in the gel, are shown in Figure 2A. Monomeric (−) strand transcripts exhibited one transition at about 20 °C, and an upper temperature transition at about 50 °C. A similar transition at about 50 °C was observed for monomeric (+) strand transcripts in addition to transitions between 29 °C and 37 °C. Moreover, distinct co-existing structures could be detected especially at the lowest temperature of the gradient. Thus, mASBVd (+) transcripts adopt alternative folds at lower temperatures whereas only one major electrophoretic profile is observed for the mASBVd (−) transcripts at all temperatures. 

In order to allow the generation of higher-order structures including tertiary interactions, mASBVd transcripts of both polarities were subjected to TGGE in buffer containing 20 μM magnesium acetate (Figure 2B). Under these conditions, mASBVd (−) transcripts migrated as a dominant single band with some weak transitions between 32 °C and 43 °C and an additional faint transition at about 51 °C. Addition of magnesium modified the transition at the lower temperatures, however, no important modifications could be observed concerning the second transition. This is consistent with what has been previously observed when PLMVd transcripts were subjected to TGGE in the same range of magnesium concentration [12]. More importantly, the TGGE profile indicated the presence of only one major conformation for mASBVd (−) transcripts that undergoes denaturation in successive transitions in the presence of magnesium. On the other hand, in the TGGE profile of mASBVd (+) transcripts, one major band and at least two additional minor bands were distinguished which present three major transitions at about 42 °C, 50 °C and 54 °C, suggesting the presence of more than one conformation. Interestingly, the co-existing structures show very similar denaturation profiles, suggesting that although they are different, these conformers share common features.

Structures were also investigated by analyzing the melting behavior of mASBVd (+) and (−) transcripts using UV-absorbance measurements under different conditions. UV-melting analyze has been shown to be a useful method to study the extensive intramolecular self-complementarity of viroids by characterizing their thermodynamic properties and structural transitions [6]. The first derivative of the melting curve (dΔA_260_/dT) plotted *versus* temperature is shown in Figure 2C,D,E. For the monomeric transcripts of (−) polarity, a major unfolding transition gave rise to a single main peak at about 51 °C in buffer containing 150 mM KCl and 10 mM sodium cacodylate (pH 7.2) (Figure 2C). The narrowness of the transition suggests a cooperative process. On the other hand, monomeric transcripts of (+) polarity exhibited an additional transition at about 58 °C, overlapping the first transition and corresponding to the main peak (Figure 2C). This observation suggests either the presence of another RNA population with a more stable folding and/or the successive denaturations of distinct regions in the mASBVd (+) transcripts. Since Mg^2+^ is well known to influence RNA tertiary structure, its presence should result in modifying the melting profiles. However, similar melting transitions were obtained when the experiments were repeated with addition of 100 μM MgCl_2 _(Figure 2D). Under those conditions, the melting behaviors of mASBVd (+) and (−) transcripts did not seem to be influenced by Mg^2+^ concentration, showing that no tertiary interactions could be detected by this analysis. When the same experiments were performed with higher salt concentration (1 M NaCl) (Figure 2E), mASBVd (+) transcript exhibited only one major peak at about 68 °C that almost completely overlaps a minor peak at 66 °C. These observations strongly suggest that both peaks correspond to secondary structures stabilized under such conditions. 

The analysis of RNA transcripts by TGGE and UV melting curves suggests that one conformation of the mASBVd (+) is thermodynamically more stable than the mASBVd (−) transcripts. Altogether, the results show that monomeric (−) and (+) transcripts exhibit different thermodynamic properties. Under different conditions, the (−) strands behave as expected for a population of molecules dominated by a major single conformation. In contrast, the (+) polarity strands seem to present either two co-existing structures or a single structure with two distinct fusion domains. TGGE experiments suggest that this rather corresponds to alternative conformers that are not necessarily detected with optical melting curve technique in which the resultant of the superimposition of all curves is observed. 

### 2.3. SHAPE Analysis Suggests the Existence of a Kissing-Loop Interaction in the (−) Strands

**Figure 3:**
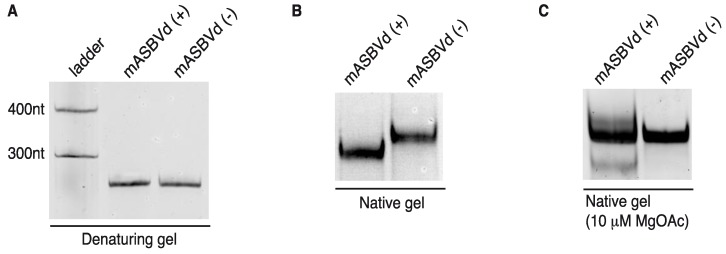
Electrophoretic mobility of mASBVd RNAs. The mASBVd (+) and mASBVd (−) transcripts were separated on 8% PAGE gels in 1× TBE buffer under denaturing (**A**) and native (**B**) conditions. The RiboRuler^TM^ low range RNA ladder (Fermentas, Thermo Fisher Scientific Inc., Waltham, MA, USA) was loaded in parallel with mASBVd (+) and (−) into the denaturing gel (A). (**C**) RNA transcripts were analyzed on 8% PAGE native gel in 1× TB buffer containing 10 μM magnesium acetate.

mASBVd (+) and mASBVd (−) RNA species were first analyzed using denaturing and native gel analyses. After heat denaturation, RNA molecules were separated on 8% denaturing polyacrylamide gel. As expected, both strands showed the same mobility under denaturing conditions confirming a similar molecular weight (Figure 3A). Indeed, the length of mASBVd (+) and mASBVd (−) strands are different by only two nt, since two guanosines have been added at the 5' terminus of mASBVd (+) to promote efficient transcription from the T7 promoter. In addition, (+) and (−) mASBVd strands were analyzed on non-denaturing 8% polyacrylamide gels (Figure 3B). Under such conditions, both the molecular weight and structure influenced the electrophoretic mobility of the RNA molecules. Before loading, the RNAs were heat denatured, and then slowly renatured in the presence of 150 mM KCl to allow them to fold into their most stable native structure. A significant difference in migration between the two strands was observed (Figure 3B, NB: the same migration profiles were observed upon renaturation in either 150 mM NaCl buffer or sterile water). Migration of mASBVd (+) is faster than mASBVd (−) suggesting that the monomeric (+) strands fold into a more compact secondary structure than the monomeric (−) strands. Similar observations have been described for the PLMVd (+) and (−) polarities; the two strands were subsequently shown to fold into different structures [12]. Then, both RNA samples were run on native 8% polyacrylamide gels using TB buffer with 10 μM magnesium acetate (Figure 3C). Under these conditions, no difference of migration could be detected between the major forms of mASBVd (+) and (−). Interestingly, two low intensity bands were also visible, above and below the major band of mASBVd (+) confirming the presence of multiple conformations, as seen in TGGE (Figure 2B). 

The structures of mASBVd (+) and mASBVd (−) RNA species were then probed using 1-methyl-7-nitroisatoic anhydride (1M7) in the presence or absence of Mg^2+^ (see Experimental Section and [27] for details on SHAPE). The modification site and intensity were determined as 1M7 dependent reverse transcription (RT) stops. Recently, this relatively new technique, that investigates both RNA structures and dynamics at single-nucleotide resolution, has been used to characterize the structure of the PLMVd as well as five members of the family *Pospiviroidae* [14,28].

**Appendix Figure A1:**
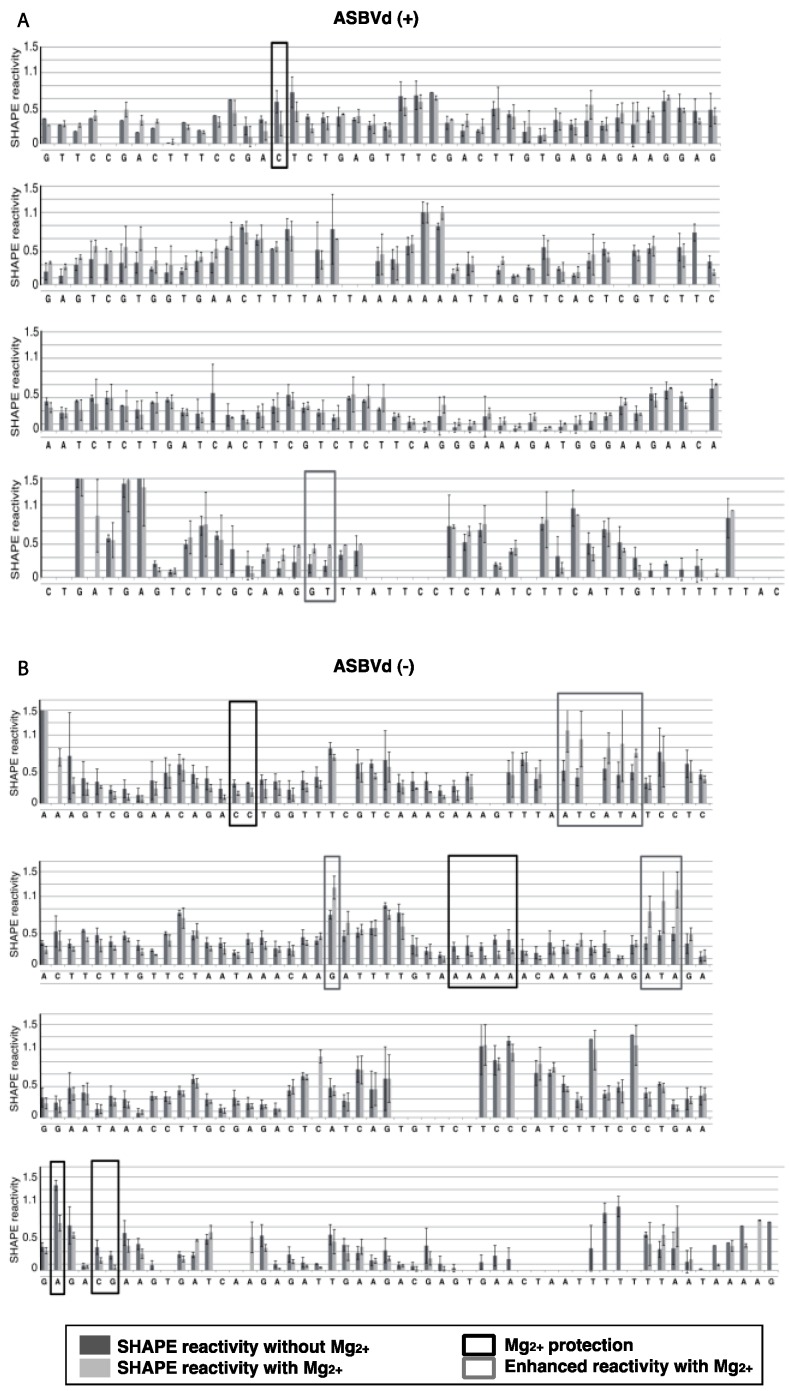
Comparison of the 1M7 reactivity profiles of ASBVd (+) (**A**) and ASBVd (−) (**B**) RNAs in the absence and in the presence of Mg^2+^. The reactivity of each nucleotide is represented in dark grey for the experiment without Mg^2+^ and in light grey in presence of 10 mM Mg^2+^. Error bars represent the standard deviation in 3 experiments. Regions boxed in black or grey highlight the nucleotides significantly (student test *p* < 0.05) under-reactive or over-reactive in the presence of Mg^2+^, respectively. The first nucleotide of each lane is numbered, 1 denotes the transcription start. Nucleotides with a negative value correspond to non-specific reverse transcriptase stops. Please note that the nucleotides at the 3' terminus which reactivity could not be determined because they are too close to the primer are not represented in these profiles although the molecules probed correspond to the full length ASBVd (+) and ASBVd (−) (64 and 44 nucleotides respectively).

The results were analyzed with ShapeFinder [29] (Appendix Figure A1), the significance of the differences observed in the presence or absence of Mg^2+^ was established using a bilateral student test (*p* < 0.05). RNA secondary structure models were first built with the help of the RNA structure software [30], using as constraints the SHAPE values obtained in absence of Mg^2+^. These models were then evaluated in the light of the probing pattern observed in presence of magnesium, and the possibility of postulating tertiary contact in this context was examined. 

**Figure 4:**
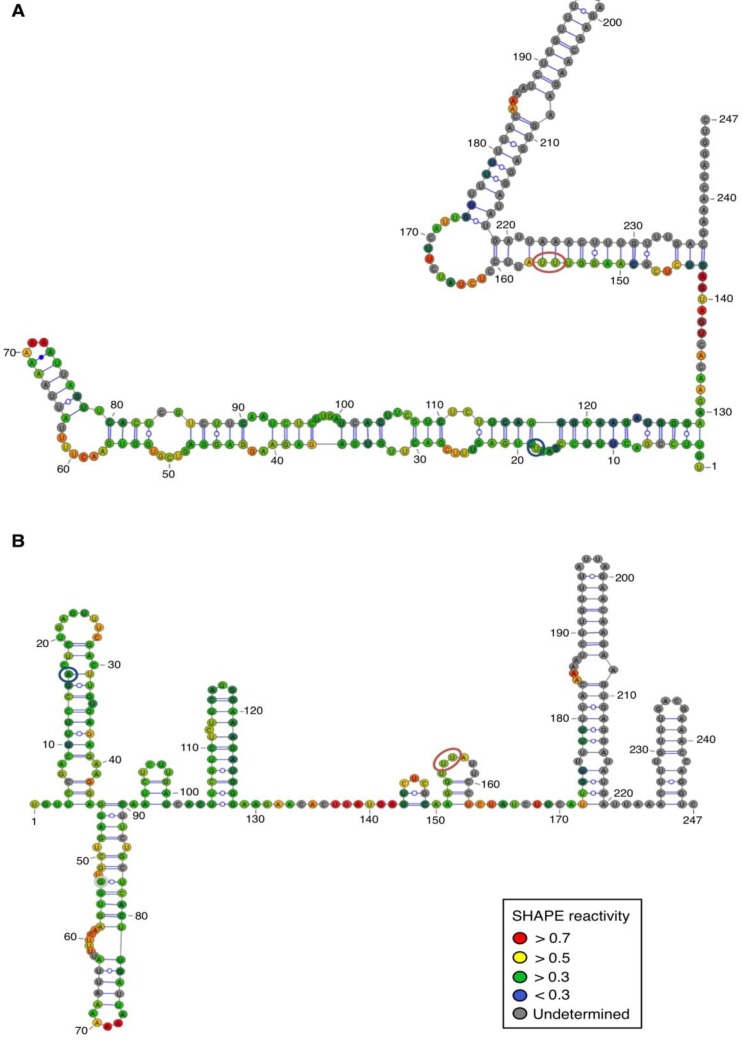
Secondary structure models of ASBVd (+) based on the SHAPE analysis. (**A**) and (**B**) are the two secondary structure models proposed for ASBVd (+). The color of the circle surrounding each nucleotide reveals their relative reactivity to 1M7 in the presence of 10 mM Mg^2+^, as mentioned in the box. High reactivity reflects single-stranded region, while paired nucleotides are not reactive. Nucleotides for which the reactivity could not be determined are shown in grey and correspond to the primer binding site, or to nucleotide right 3' to the primer for which the electrophoresis resolution is not good enough, or to RT stops. The same experiments were carried out without Mg^2+^ and differences in reactivity are represented on the scheme: nucleotides circled in red show enhanced reactivity in the presence of Mg^2+^, and those in blue are less reactive. The models were designed using VaRNA [31].

**Figure 5:**
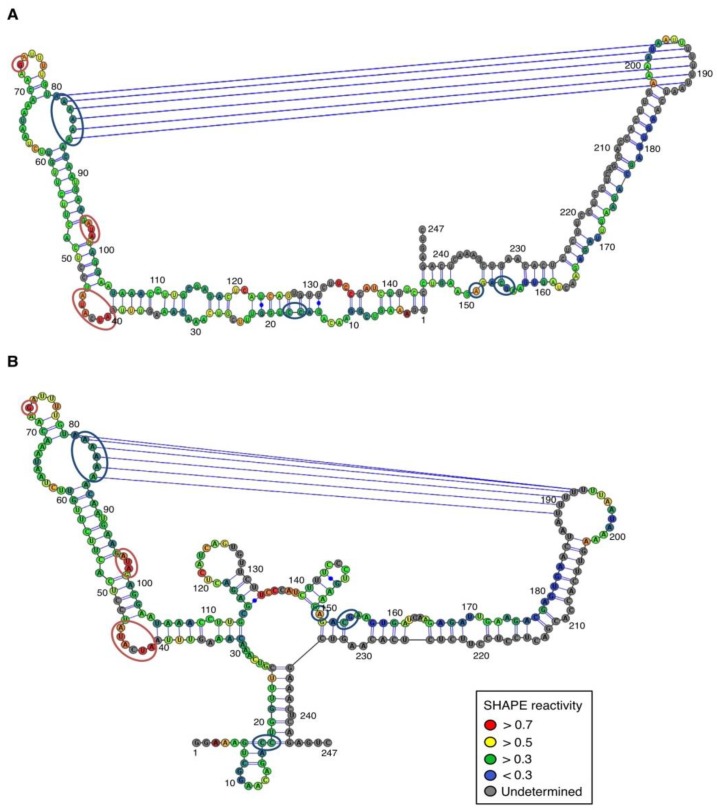
Secondary structure models of ASBVd (−) based on the SHAPE analysis. (**A**) and (**B**) are the two secondary structure models proposed for ASBVd (−). The color of the circle surrounding each nucleotide reveals their relative reactivity to 1M7 in the presence of 10 mM Mg^2+^, as mentioned in the box. High reactivity reflects single-stranded region, while paired nucleotides are not reactive. Nucleotides for which the reactivity could not be determined are shown in grey and correspond to the primer binding site, or to nucleotide right 3' to the primer for which the electrophoresis resolution is not good enough, or to RT stops. The same experiments were carried out without Mg^2+^ and differences in reactivity are represented on the scheme: nucleotides circled in red show enhanced reactivity in the presence of Mg^2+^, and those in blue are less reactive. Blue lines represent the potential kissing-loop interaction. The models were designed using VaRNA [31].

For both polarities, two models (Figure 4 and Figure 5)—a quasi-rod-like and a branched model—consistent with the reactivity profile are proposed. They are equiprobable models to date, but they may not represent different conformers. The experiments were repeated three times with good reproducibility. However, experimental noise is often noticed at 5' and 3' end, and in regions where unspecific RT stops occur (the positions before and after a stop are often variable). In addition, we noticed that the reactivity of stretches of nucleotides in specific regions showed an unusually high standard deviation. Such observations were more frequent in ASBVd (+) (see Appendix Figure A1-A nucleotides 25–26; 39–40; 43–4; 49–57; 107; 141–142; 145–146) than in ASBVd (−) (see Appendix Figure A1-B nucleotides 45; 53; 96–98; 124–125; 177). This could reflect the presence of different conformations, the relative proportion of which can vary from one experiment to another. This observation is consistent with the results obtained in native gels with Mg^2+^ (Figure 2B and Figure 3C), where alternative structures are detected, mostly in the case of the ABSVd (+). For the (+) strand, the reactivity of only two nt significantly varies upon Mg^2+^ addition; this could reflect a lack of important tertiary folding under these Mg^2+^ conditions, or that only a small portion of the molecules adopts a tertiary structure (Figure 4).

In contrast, for the (−) strand we observed a clear protection of the adenosine stretch 5'_82_AAAAA_86_3'. In addition, we noticed that positions U_190_ and U_191_, which are reactive in the absence of Mg^2+^, are detected as strong RT stops when RNA is folded in the presence of Mg^2+^ ions (Figure 5 and Appendix Figure A1). Surrounding nucleotides, U_189,_ U_192_ and U_193,_ are also detected as non–specific stops even in the absence of Mg^2+^. Since kissing-loop interactions are stabilized in the presence of divalent ions, there are difficult structures to resolve for RT as they induce stops. Our results suggest the existence of a kissing-loop interaction between nucleotides 5'_82_AAAAA_86_3' and 5'_189_UUUUU_193_3' (Figure 5).

### 2.4. Sequences Implicated in the Potential Kissing-Loop Interaction of ASBVd (−) RNAs Are Conserved in ASBVd Variants

The base pairing ability was examined in the natural sequences available. Among the 96 different sequences of ASBVd identified and listed in the Subviral RNA database [1], 90 variants (94%) encompass both the stretches of six consecutive adenines and six consecutive uracils, involved in the kissing-loop interaction of (−) ASBVd. Amongst the six exceptions identified, ASBVd.038 and the ASBVd.069 variants (accession number AF404029 and AF404060 respectively) present a stretch of five instead of six uracils, and ASBVd.052 (accession number AF404043) has a G in the A stretch and was identified in symptomless trees. Two others show important deletions and were identified in symptomless trees (*i.e*., ASBVd.083 variant, accession number AF404074, and ASBVd.082 variant, accession number AF404073). It cannot be excluded that the latter two variants, identified by RT-PCR among total RNAs extracted from leaf and flower tissue of infected avocado, represent defective viroids. The last exception is ASBVd.090 variant (accession number EU519467) in which large stretches of the sequence are significantly divergent from other ASBVd variants and notably lack both A and U repetitions. Although the existence of a putative kissing loop remains to be confirmed by mutation, the high conservation of these regions in most ASBVd sequence variants supports its existence in this particular area of ASBVd (−) strand.

In addition, it is worth noting that the postulated tertiary interaction is located near the replication initiation site of ASBVd (−) RNAs [32]. Indeed, initiation sites of strands on both polarities have been located in the terminal hairpin loops of the predicted structures [32]. Structural differences in the regions neighboring the initiation sites of both ASBVd (−) and (+) RNAs may influence their respective interactions with the RNA polymerase and/or transcription factors. ASBVd replicates via a symmetric RNA-dependant rolling-circle process, then the presence of a kissing-loop interaction could allow the ASBVd (−) strand to be efficiently transcribed into the ASBVd (+) strand. Such a speculative hypothesis might also partially explain the higher concentrations of ASBVd (+) RNAs observed in infected tissue compared to the ASBVd (−) RNA concentrations [18,19].

## 3. Experimental Section

### 3.1. Oligonucleotides

The oligonucleotides were obtained from Eurofins MWG Operon (Ebersberg, Germany) and listed below. The oligonucleotide size and ASBVd location are indicated in brackets. ASBVd1: CGGGATCCCGAAGAGATTGAAGACGAGTG (29 nt, ASBVd (−) 85-103) ASBVd2: GGAATTCCGATCACTTCGTCTCTTCAGG (28 nt, ASBVd (−) 84-65) T7-ASBVd5: TAATACGACTCACTATAGGTGTTCCGACTTTCCGACTCTG (40 nt, ASBVd (+) 56-76) ASBVd6: GACCTGGTTTCGTCAAACAAAG (22 nt, ASBVd (+) 55-34) T7-ASBVd7: TAATACGACTCACTATAGGAAAGTCGGAACAGACCTG (37 nt, ASBVd (−) 169-188) ASBVd8: GACTCTGAGTTTCGACTTGTG (21 nt, ASBVd (−) 168-148) T7-ASBVd9: TAATACGACTCACTATAGGGATCACTTCGTCTCTTCAGG (39 nt, ASBVd (+) 154-173) ASBVd10: AAGAGATTGAAGACGAGTG (19 nt, ASBVd (+) 247-219) T7-ASBVd11: TAATACGACTCACTATAGGAAGAGATTGAAGACGAGTG (38 nt, ASBVd (−) 85-103) ASBVd12: GATCACTTCGTCTCTTCAGG (20 nt, ASBVd (−) 84-65) ASBVd13: GGTTTCGTCAAACAAAGTTTAATCATATCC (30 nt, ASBVd (+) 50-21) ASBVd14: CTCTGAGTTTCGACTTGTGAGAGAAGG (27 nt, ASBVd (−) 166-140) The T7 promoter sequence allowing the subsequent *in vitro* transcription reaction of ASBVd is underlined. 

### 3.2. Synthesis of Templates for Transcription

To produce both (+) and (−) ASBVd monomeric transcripts, encompassing the hammerhead ribozyme sequence (HHR), PCR amplification of plasmid pBdASBVd [33] using primers ASBVd1 and ASBVd2 was performed. The resulting DNA fragment was digested with *BamH1* and *EcoR1* and cloned into pBlueScript II KS (+) at the corresponding restriction sites, generating plasmid pBmASBVd-HHR. The (+) ASBVd monomer, starting at position 56 (*i.e*., at the hammerhead cleavage site) was amplified by PCR of pBdASBVd using primers T7-ASBVd5 and ASBVd6. This PCR fragment was cloned into pCRII-TOPO^®^ (Invitrogen, Life Technologies, Carlsbad, NM, USA) yielding pTmASBVd (+). The (−) ASBVd monomer, starting at position 179 (*i.e*., at the hammerhead cleavage site) was amplified by PCR of pBdASBVd using primers T7-ASBVd7 and ASBVd8. The resulting DNA fragment was cloned into pCRII-TOPO^®^ yielding pTmASBVd (−). The constructs were verified by sequencing. 

### 3.3. *In Vitro* Transcription from Plasmid Templates and Isolation of RNA Transcripts

The RNA molecules used in the studies were prepared by transcription of DNA templates with T7 RNA polymerase. The DNA templates were prepared by PCR amplification of the plasmids described above: for mASBVd-HHR (+) and (−), both containing the hammerhead ribozyme sequences, plasmid pBmASBVd-HHR was amplified with primers T7-ASBVd9/ASBVd10 and T7-ASBVd11/ASBVd12, respectively. For monomeric ASBVd (+), designated mASBVd (+), pTmASBVd (+) was amplified with primers T7-ASBVd5 and ASBVd6 and for monomeric ASBVd (−), designated mASBVd (−), pTmASBVd (−) was amplified with primers T7-ASBVd7 and ASBVd8. *In vitro* transcriptions of the PCR products were performed with purified T7 RNA polymerase at 37 °C for six hours. After one hour of DNase I (Fermentas, Thermo Fisher Scientific Inc., Waltham, MA, USA) treatment, the transcription mixes were ethanol precipitated. The pellets were resuspended in stop buffer (80% formamide, 0.03% xylene cyanol and 30 mM EDTA) and then separated by denaturing PAGE (6%, 7 M urea). The bands corresponding to the full-length transcripts were excised under UV shadowing and eluted overnight in elution buffer (0.3 M sodium acetate). The RNA concentrations were quantified by spectrophotometry at 260 nm.

### 3.4. Self-Cleavage Kinetic Analyses of Isolated RNAs

mASBVd-HHR transcripts of both polarities were synthesized *in vitro* from plasmid pBmASBVd-HHR and purified as described in above paragraph. The concentrations of mASBVd-HHR (+) and mASBVd-HHR (−) were set at 15 ng/μL in buffer containing 150 mM KCl and either 10 mM sodium cacodylate (pH 7.2) or 45 mM Tris-HCl (pH 7.5 at 25 °C). RNAs were denatured for 5 min at 65 °C and slowly cooled at 25 °C to allow renaturation. Mixes were pre-heated at the appropriate temperature and self-cleavage reactions were initiated by adding magnesium chloride at final concentrations of 20 mM, 50 mM or 100 mM. Aliquots of the reaction mixes were removed at the indicated times by addition of an equal volume of stop buffer and were heat-denatured at 65 °C for 5 min before being separated by denaturing PAGE (10%, 7 M urea). After ethidium bromide staining, the products were quantified with the ImageJ software [25]. The data were fitted to multiexponential curves with the program KaleidaGraph (Synergy Software, Reading, PA, USA). 

### 3.5. Temperature Gradient Gel Electrophoresis

mASBVd (+) and mASBVd (−) RNAs, were synthesized *in vitro* from plasmid pTmASBVd (−) and pTmASBVd (+), respectively (see Section 3.3). The RNA samples (500 ng) were denatured and renatured in 50 μL, 150 mM KCl and 10 mM of sodium cacodylate (pH 7.2) buffer. After addition of 5 μL of native loading buffer (30% glycerol, 0.03% xylene cyanol), the samples were loaded onto native gels containing 8% polyacrylamide (19:1 acrylamide/bisacrylamide) in either 0.2× TBE (Figure 2A) or 0.2× TBM buffer (TB with 20 μM MgOAc) (Figure 2B) on which a linear temperature gradient is established perpendicular to the direction of the electric field. Electrophoresis was performed with a standard TGGE system (Biometra) at 400 V during 10 min at 20 °C in order to allow the samples to penetrate into the gel. This step was followed by migration for 70 min at 250 V with the appropriate temperature gradient, 20 °C–65 °C. In brief, RNAs migrate according to their charge and their degree of compaction, which changes with temperature. Roughly, denatured RNAs are less compact and run more slowly than compactly folded RNAs. Then, if several conformations coexist at a given temperature, they are separated according to their compactness. The gels were stained with ethidium bromide.

### 3.6. Melting Experiments in Solution Monitored by UV-Absorbance

Thermal melting curves were performed on a UVIKON 940 (Kontron, Everett, MA, USA) spectrophotometer. Samples were heated at 90 °C for 2 min, and cooled slowly to 20 °C. Absorbance at 260 nm and 405 nm of the RNA (25 ng/μL), in 150 mM KCl and 10 mM sodium cacodylate (pH 7.2), was recorded while heating (0.2 °C/min) from 15 °C–90 °C using quartz cells with a 1 cm path-length. The experiments were repeated in the presence of either 100 μM MgCl_2_ or 1 M NaCl. The temperature was varied with a circulating water bath cell holder and measured with an inert glass sensor immersed in a water-filled quartz cell. The absorbance recorded at 405 nm, varied slightly in a monotonous manner independently of the temperature: no potential artifact was detected. The first derivatives of the absorbance signal (d∆A_260_/dT) are presented using the program KaleidaGraph (Synergy Software, Reading, PA, USA). UV-melting experiments were performed three times.

### 3.7. Migration of ASBVd Monomers in Denaturing and Native PAGE

For denaturing gels, one volume of stop buffer was added to 50 ng of mASBVd (+) or (−). The samples were heat denatured at 65 °C for 5 min before being separated on an 8% polyacrylamide-7 M urea gel. For native gels, 50 ng of RNA of each polarity of mASBVd were denatured for 5 min at 65 °C and rapidly cooled in an ice bath, then denatured again for 5 min at 65 °C and slowly renatured, in a buffer containing either 45 mM Tris-HCl (pH 7.5 at 25 °C) and 150 mM KCl or 10 μM magnesium acetate. 1/10 volume of native loading buffer (30% glycerol, 0.03% xylene cyanol) was added to the samples prior to loading onto an 8% polyacrylamide native gel (19:1). Native gels were run in either 1× TBE (Figure 3B) or 1× TB buffer containing 10 μM magnesium acetate (Figure 3C). The gels were stained with Sybr Gold (Invitrogen, Life Technologies, Carlsbad, NM, USA).

### 3.8. Selective 2'-Hydroxyl Acylation Analyzed by SHAPE

RNA analysis by SHAPE was performed using 1M7 as a modifying agent as previously described [20,21,22]. 1M7 selectively reacts with nucleotides whose ribose is flexible, thus revealing the unpaired nucleotides *versus* the nucleotides involved in a canonical or non-canonical interaction. However, it is important to note that this is not an absolute rule and that some “single stranded” nucleotide can be only weakly reactive, because they are partially constrained in non-canonical base pairs. Briefly, 6 pmol of *in vitro* transcribed mASBVd (+) or mASBVd (−) were resuspended in 21 μL of water, and heat- denatured for 2 min at 80 °C, then 3 μL of 10× buffer containing 400 mM HEPES pH 7.5, 1 M KCl, and either 10 mM MgCl_2_ or not were added. The samples were placed at 30 °C for 10 min, then 3 µL of 1M7 were added and the mixture was incubated at 37 °C for 5 min. Negative controls, where the chemical was replaced by DMSO, were also performed. The reaction was then precipitated in dry ice with ethanol and 5 M ammonium acetate. The RNA was then resuspended in 0.5 M ammonium acetate, ethanol precipitated in the presence of glycogen, washed with 70% ethanol and resuspended in 6 μL of nuclease-free water. Modifications were revealed by reverse transcription using RNAse H M-MLV RT (Promega, Madison, WI, USA) with primer ASBVd13 for mASBVd (+) and primer ASBVd14 for mASBVd (−) labelled at the 5' end with WellRed D2, D3, D4 (Sigma-Aldrich Saint-Louis, MO, USA), or Dy-782 (Eurofins MWG Operon, Ebersberg, Germany) fluorophores. The cDNA fragments were resolved by capillary electrophoresis (Beckman Coulter, Brea, CA, USA, CEQ 8000) and the data interpreted and analyzed using the software ‘ShapeFinder’ [29]. Profiles were compared to the profile obtained with a negative control containing the equivalent amount of buffer instead of RNAs. 

## 4. Conclusions

In conclusion, we present in this study new structures of ASBVd (+) and ASBVd (−) based on data carried out on *in vitro*-synthesized linear monomeric forms of ASBVd. Clearly, it might be different in natural conditions, however, the linear strands exist *in vivo* as a genomic version of the viroid during the rolling-circle replication cycle and the results obtained could be relevant to the determination of each polarity properties *in vivo* [28]. Here, we show clear evidences that the (+) and the (−) polarities of ASBVd exhibit biophysical characteristic differences, reflecting that the linear form of the two polarities fold into different secondary structures, probably responsible for distinct properties *in vitro* that could trigger different behavior *in vivo*. Interestingly, the (+) strands exhibit alternative conformations and self-cleave very inefficiently. Our results could reflect that the active conformation is one of these minor conformations, and that most of the molecule population has to undergo an unfavorable structural rearrangement to reach the catalytic folding. It is likely that cellular proteins, such as RNA chaperone, prevent this alternative folding, or facilitate the required rearrangement. 

By comparing the structures between PLMVd and ASBVd, it is interesting to note that for each of the viroids, the kissing-loop formed in one of the polarity strands seems to be absent in the counterpart strand [12,13,14]. However, for PLMVd, the pseudoknot is present in the (+) polarity strand whereas regarding ASBVd, the kissing-loop is formed in the (−) strand. Similarly to what is observed for PLMVd, the *in vitro* self-cleavage efficiency is not identical for the two complementary strands [34]. However, the polarities that present the higher level of self-cleavage differ between the two viroids. Consequently, it is tempting to speculate that in members of the family *Avsunviroidae*, each polarity could play a distinct role during the viroid life cycle. Previously, using modeling software only based on energy minimization, a quasi-rod-like secondary structure was predicted for ASBVd, in contrast to the branched structures described for other members of *Avsunviroidae* [10]. Here, using the experimental constraints provided by the SHAPE technology, we propose two models for both polarities, a quasi–rod-like and a branched model, showing that ASBVd could also adopt a typical structure of *Avsunviroidae* members.
